# Cultivation of “Ginseng.”

**Published:** 1860-05

**Authors:** 


*Cultivation of* "*Ginseng*."—A St. Petersburg letter of the 24th December, announces the inauguration of a scientific expedition to the Chinese frontier, under the direction of M. Maak, "to describe the south-east of the Mantchou territory to the frontiers of Coree, and examine especially the *ginseng* (a renowned medicinal plant); to study the geographical extent of the Mantchou territory, where the plant is propagated, and particularly the places where it grows naturally, and to examine and describe the plantatations of the Chinese who cultivate it." A great number of Russians propose to establish plantations of *ginseng*, as an important produce for trade with China.

